# Supplementary material for: A study about management of drugs for leprosy patients under medical monitoring: A solution based on AHP-Electre decision-making methods
Source: PLoS One. 2023 Feb 13;18(2):e0276508. doi: 10.1371/journal.pone.0276508 (PMC9924998; doi:10.1371/journal.pone.0276508)
Supplement: Attachment — Submitted filename: Response to Reviewers.pdf [file pone.0276508.s002.pdf]

Dear Editor,

We thank again the evaluations of the two reviewers regarding the article “Use of Multi-criteria Methods to Support Decision-Making in Drug Management for Leprosy Patients”. Thus, after evaluating what was indicated by the reviewers, several pertinent points were considered, which led us to a profound restructuring of the manuscript, aiming to make it even more coherent and with better results. Thus, we highlight the following points:

- The use of the analytical model AHP and TOPSIS, which was used in the initial version of the article, was rethought. After testing with other models, it was found that the hybrid use of AHP with ELECTRE II (instead of TOPSIS) generated more reliable results for leprosy drug decision making. Therefore, the new version of the paper brings results from the combination of AHP and ELECTRE II.
- With the aforementioned change in the decision model, more accurate results were obtained, thus, it was necessary to restructure the sections of the article, so the TOPSIS section was renamed as ELECTRE II (Abstract, Introduction, Related Work, Material and Methods, Case Study, Results, Conclusion).
- The title of the article "Use of Multi-criteria Methods to Support Decision-Making in Drug Management for Leprosy Patients" is consistent with the proposal, even after the modifications suggested by the reviewers and the change in the analytical model. However, the title "A Study About Management of Drugs for Leprosy Patients Under Medical Monitoring: A Solution Based on AHP-Electre Decision-Making Methods" makes the solution of the study even more evident.
- With the amount of adjustments mentioned above, added with the indication of the Reviewers, comments, of English revision, it was necessary the invitation of one more author ADRIANO MADUREIRA DOS SANTOS, who was responsible for generating new results and performing a complete revision of the English writing. Therefore, this author was added to the list of authors.

The authors of the paper (PONE-D-21-36593R1) would like to express their gratitude for the valuable comments and advice of the reviewers; we believe that these changes have improved the quality and readability of the paper.

1. The captions (description at figures) for figures are missing.

   Authors’ response: The manuscript has been adjusted, adding the correct description and caption for the figures.

2. Explaining the contents of Table 1 better and writing the sentence ``The calculation of CR is given by Eq2.'' before Eq.2.

Authors’ response: The description in table 1 of the AHP section has been adjusted as requested. (Material and Methods section, AHP subsection, page 7)

3. Explaining the prioritization patient process using (AHP) and (TOPSIS), i.e. illustrative of at least one case of a patient in the Case study.

   Authors’ response: All the items mentioned have been corrected, illustrating the patient examples in the case study (Material and Methods section, Case Study subsection, page 8).

4. Clarifying calculation of the Eigen value of the matrix, the corresponding Eigenvector, and Main Value, (Table 3 and Table 4).

   Authors’ response: The table 3 and 4 corresponding Eigenvector items were correctly adjusted. (Results section, page 10).

5. There are several mistakes all over the text. Some of them have been corrected in the Manuscript Draft (see, attachment file).

   Authors’ response: We have carried out an extensive spelling check, removing redundancies and typos throughout the text.

6. In “Abstract”, the period of study stated as “… between 2015 and 2020”, but in the main body this state as “… 2016-2020”. Line 54, line 158, and line 262.
   Authors’ response: The cited excerpt from the abstract was adjusted according to the request (abstract, page 1).

7. There is a series of superscript numbers and abbreviations in the text which must be modified in accordance with the format approved by the journal (e.g line 8, line 127, “…as per”).
   Authors’ response: We have carried out an extensive spelling check, correcting the mentioned items and checking the journal format.

8. The order of the references in the text is incorrect. For example, after the reference 8, the reference 10 comes, and after that, reference number 30, 26 and ...
   Authors’ response: New references have been added to the manuscript. Thereon, the order of the references were adjusted according to the request.

9. Under the "Related Work", the authors stated FIVE related study (ref 13-17). Please discuss more study, For example, the following studies:
   Authors’ response: All references recommended by the reviewers were added in the related work section of the manuscript (Related work, page 3).

- Gilabert-Perramon, A., Torrent-Farnell, J., Catalan, A., Prat, A., Fontanet, M., Puig-Peiró, R., . . . Badia, X. (2017). “Drug evaluation and decision making in catalonia: development and validation of a methodological framework based on multi-criteria decision analysis (MCDA) for orphan drugs”. International Journal of Technology Assessment in Health Care, 33(1), 111-120. doi:10.1017/S0266462317000149.
- A Moosivand, M Rangchian, L Zarei, F Peiravian, G Mehralian, ... (2021). “An application of multi-criteria decision-making approach to sustainable drug shortages management: evidence from a developing country”. Journal of Pharmaceutical Health Care and Sciences 7 (1), 1-11.
- Vinayak Vishwakarma, Chandra Prakash, Mukesh Kumar Barua.(2016).”A fuzzy-based multi criteria decision making approach for supply chain risk assessment in Indian pharmaceutical industry”. International Journal of Logistics Systems and Management. Vol. 25, No. 2. pp 245-265.
- Jason C. Hsu , Jia-Yu Lin, Peng-Chan Lin, Yang-Cheng Lee (2019). “Comprehensive value assessment of drugs using a multi-criteria decision analysis: An example of targeted therapies for metastatic colorectal cancer treatment”. PLOS ONE. https://doi.org/10.1371/journal.pone.0225938.

10. The figure’s number in the manuscript and PDF provided for review does not match.

    Authors’ response: The figure’s number order in the PDF format in the manuscript were adjusted according to the review.

11. There are occasional minor typos in the manuscript. For example, “in homogeneous” instead to “inhomogeneous”.

    Authors’ response: We have carried out an extensive spelling check, removing redundancies and typos throughout the text.

12. In line 265-266, the authors stated that “…three factors: (a) the low quantity of medications distributed in the basic health units; (b) the in homogeneous distribution of medications; (c) patient evasion.” I did not understand the concept of "patient evasion ". I think here there is a need to add an explanation.

    Authors’ response: The cited sentence was adjusted in the manuscript, improving the explanation of items such as "patient evasion" that can confuse the reader (Material and Methods section, Case Study subsection, page 8).

13. What is the meaning of "the responsible agencies" in line 269?

    Authors’ response: The mentioned expression was removed, with the aim of improving the understanding of the manuscript.

14. In line 273-274, it is stated that “It is worth noting that 273 only New Cases are patients confirmed for Leprosy”, which does not conform to the definition provided for “Relapse” in line 270.
    Authors’ response: The manuscript has been adjusted, correcting the amount expressed in the text relapse cases (Results section, page 9).

15. The patient classification must be the same throughout the manuscript. At present, for example, for “Relapse“ in the text and figures, similar words such as recurrence etc. have been used, which need to be uniform.
    Authors’ response: The manuscript has been adjusted, meeting all reviews in relation to the items mentioned (Material and Methods section, Case Study subsection, page 9).

16. The line 275 to 282 does not make sense. The numbers and percentages provided do not match. The sum of 18% and 43% is equal to 61%, which is equal to 616 out of 1010 patients.
    Authors’ response: The percentage of new cases and relapse was adjusted in the manuscript, meeting the requested correlation (Material and Methods section, Case Study subsection, page 9).

17. The authors have stated in line 286-288 that “This condition is a function established by professionals of the field, who take into consideration several clinical, laboratory and neurological attributes to define the treatment burden.” Please provide more information about the composition and number of experts participating in the study.
    Authors’ response: An explanation of the types of experts and people who participated in the study was added to the manuscript. This condition improved the explanation of the scenarios and the attributes of the experiment's inputs (Material and Methods section, Case Study subsection, page 9).

18. It is expected that few of the readers of this journal are familiar with the analysis method applied by the authors. In order to make it easier for novice readers to understand the contents, please provide additional explanations on the meaning of the weights in the tables and the interpretation of the results (numerical values).
    Authors’ response: A new explanation about the meaning of the weights in the tables has been added to results, in order to facilitate the explanation of the input data in the manuscript (Results section, page 10).

19. The authors do not discuss the implications of their findings. Doing so would make the study more interesting to the reader and actually support potential policy changes. I suggest starting the discussion by repeating the objective of the study and followed by each main finding that answered each specific research questions.

Authors’ response: The text underwent an extensive spelling check, where new items were added in its structure with the aim of improving its presentation and its discussion of the contributions.

20. Overall the discussion section need to be added, and must be written exactly.

Authors’ response: The text underwent an extensive spelling check. The manuscript underwent an extensive revision in its structure, where new elements were added and a discussion was made of certain of the main contributions of the text, as the benefits of a study of this nature for the academy and on the social impact of the study

We are grateful to the reviewers for all their advice and will be very pleased to add any further information if this is required.

Thank you for your kind attention.

Yours sincerely,
