# Supplementary material for: A study about management of drugs for leprosy patients under medical monitoring: A solution based on AHP-Electre decision-making methods
Source: PLoS One. 2023 Feb 13;18(2):e0276508. doi: 10.1371/journal.pone.0276508 (PMC9924998; doi:10.1371/journal.pone.0276508)
Supplement: Attachment — Submitted filename: Dear Associate Editor.docx [file pone.0276508.s001.docx]

**Dear editor of PLOS ONE,**

The subject is "Use of Multi-criteria Methods to Support Decision-Making in Drug Management for Leprosy Patients"(PONE-D-21-36593), which is interesting and applicable. However, it will be suitable for publication if its shortcomings improve. In my view, the manuscript has several shortcomings as following;

1- In “Abstract”, the period of study stated as “… between 2015 and 2020”, but in the main body this state as “… 2016-2020”. Line 54, line 158, and line 262.
2- There is a series of superscript numbers and abbreviations in the text which must be modified in accordance with the format approved by the journal (e.g line 8, line 127, “…as per”).
3- The order of the references in the text is incorrect. For example, after the reference 8, the reference 10 comes, and after that, reference number 30, 26 and ...
4- Under the "Related Work", the authors stated FIVE related study (ref 13-17). Please discuss more study, For example, the following studies:

- Gilabert-Perramon, A., Torrent-Farnell, J., Catalan, A., Prat, A., Fontanet, M., Puig-Peiró, R., . . . Badia, X. (2017). “Drug evaluation and decision making in catalonia: development and validation of a methodological framework based on multi-criteria decision analysis (MCDA) for orphan drugs”. International Journal of Technology Assessment in Health Care, 33(1), 111-120. doi:10.1017/S0266462317000149
- A Moosivand, M Rangchian, L Zarei, F Peiravian, G Mehralian, ... (2021). “An application of multi-criteria decision-making approach to sustainable drug shortages management: evidence from a developing country”. Journal of Pharmaceutical Health Care and Sciences 7 (1), 1-11
- Vinayak Vishwakarma, Chandra Prakash, Mukesh Kumar Barua.(2016).” A fuzzy-based multi criteria decision making approach for supply chain risk assessment in Indian pharmaceutical industry”. International Journal of Logistics Systems and Management. Vol. 25, No. 2. pp 245-265
- Jason C. Hsu , Jia-Yu Lin, Peng-Chan Lin, Yang-Cheng Lee (2019). “Comprehensive value assessment of drugs using a multi-criteria decision analysis: An example of targeted therapies for metastatic colorectal cancer treatment”. PLOS ONE. <https://doi.org/10.1371/journal.pone.0225938>.

5- The figure’s number in the manuscript and PDF provided for review does not match.
6- There are occasional minor typos in the manuscript. For example, “in homogeneous” instead to “inhomogeneous”.
7- In line 265-266, the authors stated that “…three factors: (a) the low quantity of medications distributed in the basic health units; (b) the in homogeneous distribution of medications; (c) patient evasion.” I did not understand the concept of "patient evasion ". I think here there is a need to add an explanation.
8- What is the meaning of "the responsible agencies" in line 269?
9- In line 273-274, it is stated that “It is worth noting that 273 only New Cases are patients confirmed for Leprosy”, which does not conform to the definition provided for “Relapse” in line 270.
10- The patient classification must be the same throughout the manuscript. At present, for example, for “Relapse“ in the text and figures, similar words such as recurrence etc. have been used, which need to be uniform.
11- The line 275 to 282 does not make sense. The numbers and percentages provided do not match. The sum of 18% and 43% is equal to 61%, which is equal to 616 out of 1010 patients.
12- The authors have stated in line 286-288 that “This condition is a function established by professionals of the field, who take into consideration several clinical, laboratory and neurological attributes to define the treatment burden.” Please provide more information about the composition and number of experts participating in the study.
13- It is expected that few of the readers of this journal are familiar with the analysis method applied by the authors. In order to make it easier for novice readers to understand the contents, please provide additional explanations on the meaning of the weights in the tables and the interpretation of the results (numerical values).
14- The authors do not discuss the implications of their findings. Doing so would make the study more interesting to the reader and actually support potential policy changes. I suggest starting the discussion by repeating the objective of the study and followed by each main finding that answered each specific research questions.
15- Overall the discussion section need to be added, and must be written exactly.
